# Muscovy duck reovirus σNS protein triggers autophagy enhancing virus replication

**DOI:** 10.1186/s12985-017-0722-8

**Published:** 2017-03-14

**Authors:** Yijian Wu, Longping Cui, Erpeng Zhu, Wuduo Zhou, Quanxi Wang, Xiaoping Wu, Baocheng Wu, Yifan Huang, Hung-Jen Liu

**Affiliations:** 1 College of Animal Science, Fujian Agriculture and Forestry University, Fuzhou, 350002 People’s Republic of China; 2 Fujian Key Laboratory of Traditional Chinese Veterinary Medicine and Animal Health (Fujian Agricultural and Forestry University), Fuzhou, 350002 People’s Republic of China; 3 Institute of Molecular Biology, National Chung Hsing University, Taichung, 402 Taiwan; 4 Rong Hsing Research Center for Translational Medicine, National Chung Hsing University, Taichung, 402 Taiwan; 5 Agricultural Biotechnology Center, National Chung Hsing University, Taichung, 402 Taiwan

**Keywords:** Muscovy duck reovirus, Autophagy, LC3-II, Viral replication, σNS nonstructural protein

## Abstract

**Background:**

Muscovy duck reovirus (MDRV) causes high morbidity and mortality in Muscovy ducklings at 10 days old and can persist in an infected flock until the ducklings of 6 weeks old. It shares common physicochemical properties with avian reovirus (ARV) and differs in coding assignment and pathogenicity. The ARV p17 protein has been shown to trigger autophagy via activation multiple signaling pathways, which benefits virus replication. Since MDRV lacks the p17 protein, whether and how MDRV induces autophagy remains unknown. The aim of this study was to explore whether MDRV induces autophagy and which viral proteins are involved in MDRV-induced autophagy.

**Methods:**

The autophagosome-like structures in MDRV-infected cells was observed under transmission electron microscopy. MDRV-induced autophagy was examined by analyzing the LC3-II level and phosphorylated form of mammalian target of rapamycin (mTOR) by Western blot assays. The effects of 3-methyladenine, rapamycin, chloroquine on viral yields were measured with quantitative(q) real-time reverse transcription (RT)-polymerase chain reaction (PCR) and 50% tissue culture infective dose (TCID_50_) assays, respectively. Additionally, to determine which viral protein is responsible for MDRV-induced autophagy, both p10.8- and σNS-encoding genes of MDRV were cloned into the pCI-neo-flag vector and transfected into DF-1 cells for detection of LC3-II.

**Results:**

The typical double-membrane vesicles containing cytoplasmic inclusions were visible in MDRV-infected immortalized chicken embryo fibroblast (DF-1) cells under transmission electron microscopy. Both primary Muscovy duck embryo fibroblasts (MDEF) and DF-1 cells infected with MDRV exhibited a significant increased levels of LC3-II accompanied with downregulation of phosphorylated form of mTOR, further confirming that MDRV is capable of inducing autophagy. Autophagy could be suppressed by 3-methylademine and induced by rapamycin and chloroquine. Furthermore, we found that σNS induces an increased levels of LC3-II, suggesting that the MDRV σNS protein is one of viral proteins involved in induction of autophagy. Both qRT-PCR and TCID_50_ assays showed that virus yield was increased in rapamycin treated DF-1 cells following MDRV infection. Conversely, when infected cells were pretreated with chloroquine, virus yield was decreased.

**Conclusions:**

The MDRV σNS nonstructural protein is responsible for MDRV-induced autophagy and benefits virus replication.

## Background

Muscovy duck reovirus (MDRV) infects 4 to 45-day-old Muscovy ducklings and causes "liver white spot disease" with a high morbidity and mortality. This acute infectious disease is characterized clinically by soft foot, diarrhea, stunted growth and white necrotic foci in liver and spleen [1–3], resulting in huge economic losses in Muscovy duck production. As a member of the family *Reoviridae*, genus *Orthoreovirus*, MDRV shares common physicochemical properties and morphological characteristics with other reoviruses isolated from chicken, commonly named avian reovirus (ARV) [4, 5]. Its genome consists of ten segments of double-stranded RNA (dsRNA) which can be divided into three size classes: three large (L1-L3), three medium (M1-M3) and four small (S1-S4) segments based on their electrophoretic mobility [4]. MDRV differs from ARV in S1 and S4 segments and lacks p17- and p10-ecoding genes [4–6]. The MDRV σNS and p10.8 nonstructural proteins are encoded by the S3 genome segment and the first open reading frame (ORF) of bicistronic S4 genome segment, respectively while the ARV σNS and p10 proteins are encoded by the S4 gene and first ORF of tricistronic S1 gene [5, 6]. Apart from the differences in coding assignment, MDRV also shows more clinical virulence than ARV. In the last few years, new types of MDRV cause hemorrhagic-necrotic lesions in the liver and spleen of sick birds and increase morbidity and mortality [7–9]. Therefore, it is of great importance to elucidate the pathogenesis and to propose efficient ways for prevention and control of MDRV infection.

Autophagy is an evolutionarily conserved life process in eukaryotic cells to degrade intracellular substrates and participates in multiple physiological processes, and maintains cellular homeostasis [10]. Cytoplasmic biological macromolecules and damaged organelles produced by stress, cancer and infectious diseases were removed during this process that includes sequestration of the substrates into autophagosomes, fusion of the vesicles with lysosomes, and subsequent degradation of its contents and inner membrane [10]. Microtubule associated protein 1 light chain 3 (LC3) exists in two different forms. The free form of LC3 type I (LC3-I) is cytoplasmic and the phosphatidylethanolamine-conjugated form of LC3 type II (LC3-II) locates at autophagosomal membranes. During the autophagy process, LC3-I is converted into LC3-II, which is essential for autophagosome formation [11]. Given the increased LC3-II and its co-localization properties with autophagosomal membranes, LC3-II-related assays are frequently used for detection of autophagy. Autophagy is regulated by several cellular signaling pathways, one of which involves the class I phosphoinositide 3-kinase (PI3K) and mammalian target of rapamycin (mTOR) is well-studied, which negatively regulate autophagy, while the class III PI3K/Beclin-1 pathway positively regulates autophagy [12, 13]. In addition, many other factors such as p53, phosphatase and tensin homolog deleted on chromosome ten (PTEN) and adenosine monophosphate-activated protein kinase (AMPK), and PKR-eIF2α have been demonstrated to regulate the formation of autophagosome [14]. Moreover, complicated crosstalk between apoptosis and autophagy has been proposed [15, 16]. Many intermediate regulators such as Beclin-1, Bcl-2 and caspase-8 have been demonstrated to regulate both apoptosis and autophagy [17–19]. Considering the characteristics of autophagy process and viral evolution, many viruses utilize autophagy to promote virus replication [14, 20–22]. A recent study has shown that ARV triggers autophagy to increase its own replication via activation multiple signaling pathways [14, 22]. A recent study by Chi et al. suggested that the ARV p17 nonstructural protein triggers autophagy via activation of PI3K/AKT/mTOR, PTEN, AMPK, and PKR-eIF2α signaling pathways [14]. Several reports also suggest that the p17 protein targeting to the nucleus is critical for induction of autophagy [23, 24]. Nevertheless, because of the lack of p17-corresponding gene, this study aimed to explore whether and how MDRV induces autophagy. In the present study, we uncovered that the MDRV YB strain triggers autophagy in both immortalized chicken embryo fibroblast (DF-1) and primary Muscovy duck embryo fibroblasts (MDEF) cells and thus benefits virus replication. Our findings also reveal that the MDRV σNS protein is involved in MDRV-induced autophagy. The present study provides first evidence of how σNS protein of MDRV affects the process of autophagy for its replication and highlights the potential role of autophagy in pathogenesis of MDRV.

## Methods

### Cells, virus and plasmid

DF-1 cells used in this study were obtained from Cell bank of Chinese academy of sciences in Shanghai. MDEF cells derived from 13-day-old specific-pathogen-free embryonated eggs were prepared using a routine method. These cell lines were maintained in Dulbecco's Modified Eagle's Medium (DMEM) supplemented with 10% fetal bovine serum (FBS) and grown at 37 °C in a 5% CO_2_ humidified incubator. Cells were seeded 1 day before each experiment in 6-cm cell culture dishes with 1 × 10^6^ cells. All cells were cultured in serum-free medium for 2 h followed by refreshing the medium containing 5% of FBS and continuing growth until cell confluence reached about 75%. MDRV YB strain was isolated and stored by our laboratory, and propagated in MDEF and purified essentially as previously described [3, 25]. The pCI-neo expression vector with an N-terminal flag sequence was constructed by Professor Liu's laboratory, National Chung Hsing University.

### Reagents and antibodies

Protein assay kit and Trizol reagent were purchased from TransGen Co. (Beijing, China). Chloroquine was purchased from Sigma-Aldrich Co. (St. Louis, USA). Rapamycin, 3-methyladenine and pre-stained protein marker were purchased from Thermo Fisher Scientific (Rockford, USA). Reverse transcription and SYBR^@^Green I assay kit were from TaKaRa Co. (Dalian, China). Polyclonal antibodies against MDRV p10.8 and σNS were our laboratory stock [26]. Rabbit anti-LC3 polyclonal antibody was purchased from Sigma-Aldrich Co. Rabbit anti-mTOR, rabbit anti-phosphorylated p-mTOR (Ser2448), rabbit anti-β-actin polyclonal antibodies and horseradish peroxidase (HRP) conjugated goat anti-rabbit IgG (H + L) antibodies were purchased from Cell Signaling Technology (Danvers, USA).

### Transmission electron microscopy

Transmission electron microscopy (TEM) was performed for autophagosomes observation. DF-1 cells were mock-infected or infected with MDRV YB strain at multiplicity of infection (MOI) of 1 for 24 h. Medium was removed and cells were washed with PBS, and the collected samples were sent to Fujian Medical University for preparation and observation of electron microscope ultrathin sections. Briefly, the collected samples were then stabilized with 2% paraformaldehyde-2.5% glutaraldehyde (Polysciences Co., Pennsylvania, USA) in 0.1 mol/L phosphate buffer at 4 °C overnight, and subsequently washed three times (Ten minutes per time) in phosphate buffer, followed by post-fixation in 1% osmium tetroxide (Polysciences Inc.) for 1 h at room temperature. The samples were dehydrated using a graded series of ethanol (50–100%) and finally embedded in Eponate 12 resin (Ted Pella Inc., Redding, CA). Then the cells were stained with 1% aqueous uranyl acetate (Ted Pella Inc.) for 1 h at room temperature, and then structures of autophagosome-like vesicles were viewed under H-7650 transmission electron microscope (Hitachi, Japan) at 80 kV.

### Virus infection and drug treatments

In order to explore that whether MDRV YB strain induces autophagy, DF-1 cells were seeded in 6-cm cell culture dishes with 1 × 10^6^ cells overnight and then infected with MDRV at an MOI of 1. After a 2 h absorption, supernatants were removed and the cells were washed three times with phosphate buffer saline (PBS) and then cultured in DMEM supplemented with 2% FBS. Apart from MDRV infection, rapamycin, chloroquine, 3-methyladenine treatments as well as inactivated MDRV- and mock-infected cells were collected for analyzing induction of autophagy. Cells were pretreated with the above drugs for 1 h followed by MDRV infection at an MOI of 1. Heat inactivation of MDRV was done by heating for 1 h at 70 °C. Similar treatments were performed on MDEF cells. Samples of each treatment were collected at different time points and then subjected to Western blot analysis of autophagy-related proteins.

### Plasmid construction and transfection

To further analyze that whether the autophagy induced by MDRV is associated with MDRV nonstructural proteins, the p10.8- and σNS-encoding genes of MDRV were amplified by reverse transcription-polymerase chain reaction (RT-PCR) with the respective primers (Table 1). PCR products were purified with gel extraction kit (Omega, Colorado, USA). The purified products were digested with the respective restriction enzymes for 2 h. The digested PCR products were cloned into the corresponding sites in the pCI-neo-flag vector. Recombinant plasmids were transformed into *Escherichia coli* DH5α chemically competent cells (China) and the plasmids were extracted with endotoxin-free Plasmid Mini Kit (Omega). For transfection, DF-1 cells were seeded into 6-well plates. At about 80% confluence, cells were transfected with respective constructs by using ViaFect™ transfection reagent according to the manufacturer’s instructions (Promega, Wisconsin, USA). In this study, ratio of ViaFect™ transfection reagent (μL): DNA (μg) was 2–3: 1, and the amount of plasmid DNA was 4 μg per well.

**Table 1:** Primers used for amplification of the p10.8- and σNS-encoding genes of MDRV

Gene	Primers	Primer sequence (5′-3′)	Expected size
p10.8	P1F	CGAATTCCATGGCTGATGCTTTTGAAGT (*EcoR* I)	288 bp
	P2R	TTGCGGCCGCCTAGTTAGATCCCGAGAG (*Not* I)	
	P3F^a^	CGTGTCCTGTCGGTCTTAGC	125 bp
	P4R^a^	TGAAGGTGGTATTCGTTCCAG	
σNS	P5F	CCACGCGTACCCATGGACAACACCGT (*Mlu* I)	1104 bp
	P6R	TCCTGTCGACCTACGCCATCCTAGCTG (*Sal* I)	
GAPDH	P7F^a^	TGCTAAGCGTGTCATCATCT	187 bp
	P8R^a^	AGTGGTCATAAGACCCTCCA	

*F* forward primer, *R* reverse primer. ^a^Primer pairs P3F/P4R and P7F/P8R used in qRT-PCR. The restriction sites in the primers are underlined

### Electrophoresis and Western blot assays

Cells were seeded in 6-well-dish 1 day before treatment with drugs or infected/ transfected with virus/plasmid as described above. Collected cells were washed with 1X PBS and lysed with RIPA buffer containing 1 mM proteinase inhibitor phenylmethanesulfonyl fluoride (PMSF) for 30 min on ice. After centrifugation of lysates at 13,000 rpm for 15 min at 4 °C, the supernatant was transferred to a new Eppendorf tube for determination of the concentration of soluble protein with the Bio-Rad Protein Assay (Bio-Rad Laboratories, USA). Assays were performed according to the manufacturer’s protocol. Equal amounts of samples were mixed with 2.5X Lammeli loading buffer and boiled for 10 min in a water bath. The proteins were separated in a 10% sodium dodecyl sulphate (SDS)-polyacrylamide gel electrophoresis (PAGE) gel and transferred to the nitrocellulose membranes using a Trans-Blot cell (Bio-Rad Laboratories, Inc., USA). The membranes were blocked for 2 h at room temperature with 5% (m/v) non-fat dry milk in Tris buffer, 0.05% (v/v) Tween 20, pH 7.4 (TBST), and washed three times in TBST, and then subjected to proper dilution of first antibodies (Rabbit anti-LC3, rabbit anti-p-mTOR Ser2448 and rabbit anti-β-actin antibodies) overnight at 4 °C, respectively. The membranes were rinsed with TBST for three times, followed by 1 h-incubation at 37°Cwith HRP-conjugated goat anti-rabbit immunoglobulin antibodies (Shifeng Biotech Co., Shanghai, China) diluted 1:5000 in TBST. The results were detected on film (GE Healthcare Life Sciences) after the membrane incubation with enhanced chemiluminescence reagent (ECL plus) (Amersham Biosciences, UK). The intensity of target proteins was calculated using the program gel-pro analyzer 4 (Media Cybernetics, USA).

### Real-time quantitative(q) reverse transcription (RT)-polymerase chain reaction (﻿RT-PCR)

To determine the effects of MDRV-induced autophagy on virus replication, DF-1 cells were plated in 6-cm cell culture dishes. Cells were infected with MDRV at an MOI of 1 for 24 h. In rapamycin, chloroquine, and 3-methyladenine treatments, cells were pretreated with these drugs for 1 h followed by MDRV infection at an MOI of 1 for 24 h. Samples of each group were collected at different time points followed by real-time quantitative RT-PCR (qRT-PCR). Briefly, total RNA was extracted from each group with TransZol Up Plus RNA Kit (TransGen, Beijing, China) according to the manufacturer’s instructions. cDNA was synthesized with PrimeScript™ RT Master Mix (TaKaRa, Dalian, China), and qPCR was performed with SYBR Premix DimerEraser™ (TaKaRa, Dalian, China) on an Applied Biosystems 7500 real-time PCR cycler (Applied Biosystems, CA, USA). The primers for qRT-PCR were shown in Table 1. PCR was performed with an initial incubation of 30 s at 95 °C, followed by amplification for 40 cycles (95 °C for 5 s, 57 °C for 30s and 72 °C for 34 s). Each sample was run in triplicate. The specificity of amplification was confirmed by melting curve analysis. The expression levels of the p10.8 gene was analyzed by 2-ΔΔCt method and normalized relative to that of the housekeeping GAPDH gene.

### Determination of virus titers

To further evaluate the effect of autophagy regulation on virus yield, DF-1 cells were pretreated with rapamycin or chloroquine for 1 h followed by infection with MDRV at an MOI of 1. When obvious CPE was observed, supernatants of each treatment were collected for TCID_50_ assay to determine virus titers with Reed-Muench calculation [27].

### Statistical analysis

The data were analyzed with Student’s t-test and SPSS V17.0 software (SPSS Inc., Chicago, USA). *P* values of less than 0.05 were considered to be statistically significant.

## Results

### Autophagosome-like structures are formed in MDRV-infected DF-1 cells

**Fig. 1:**
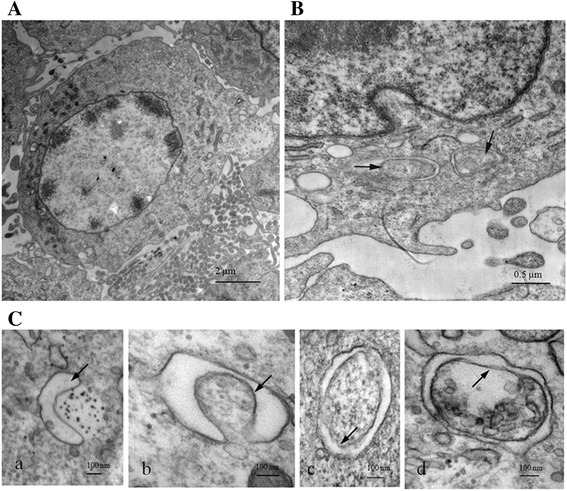
MDRV infection increases the formation of autophagosome-like vesicles in DF-1 cells. DF-1 cells were mock-infected or infected with MDRV YB strain at an MOI of 1 for 24 h, and then prepared into ultrathin sections and observed under transmission electron microscopy. **a** shows the normal morphology of DF-1 cells and autophagosome-like structure with typical double-membrane vesicles containing cytoplasmic inclusions were indicated by as *arrows* in (**b**) and (**c**). Panels *a*, *b*, *c* and *d* in (**c**) indicate different stages of autophagosome-like structures with a higher resolution. Scale bars were presented in the lower right corner

As compared to ARV, MDRV shows more mortality rate to host and causes great economic losses in Muscovy duck production. The high pathogenicity of MDRV mainly depends on the interaction between virus and host. To determine whether MDRV is capable of inducing autophagy in cultured cells, transmission electron microscopy was carried out on MDRV-infected or mock-infected DF-1 cells. As shown in Fig. 1, MDRV-infected DF-1 cells exhibited typical double-membrane vesicles (autophagosome-like) (Fig. 1b-c) containing cytoplasmic inclusions that were not present in the mock group (Fig. 1a).

### MDRV induces autophagy in DF-1 and MDEF cells via suppression of mTOR phosphorylation

**Fig. 2:**
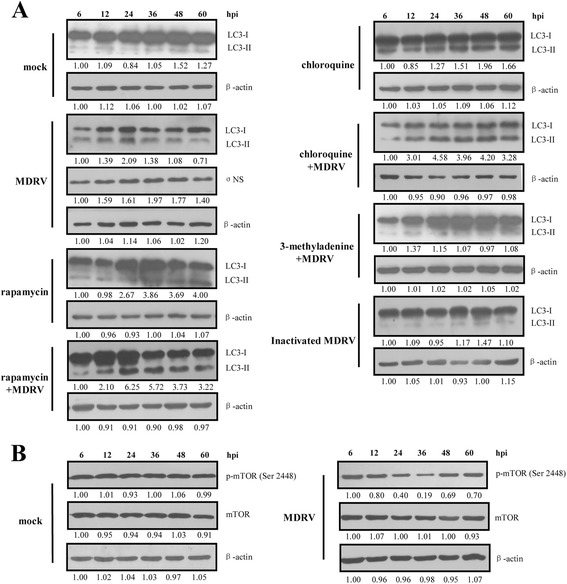
The YB strain of MDRV induces autophagy in DF-1 cells with concomitant suppression of mTOR phosphorylation. DF-1 cells were cultured in 6-well plates, and infected with MDRV at an MOI of 1. Samples were collected at indicated time points and analyzed with Western blot assays using specific antibodies against of LC3 I/II (**a**), mTOR (**b**), p-mTOR (Ser 2488) (**b**), and β-actin (**a**, **b**). DF-1 cells were pretreated with rapamycin, chloroquine, 3-methyladenine for 1 h, respectively, followed by MDRV infection at an MOI of 1. Heat inactivated MDRV was achieved by heating for 1 h at 70 °C. The activation and inactivation folds in each treatment indicated below each lane were normalized against those at 6 h. The proteins levels were normalized to those for β-actin. The levels of indicated proteins at 6 h were considered 1 fold. Similar results were obtained in three independent experiments

To further confirm that autophagy is induced in MDRV-infected DF-1 cells, LC3-II, an important hallmark of autophagy induction, was detected by immunoblot assays. All of the protein samples were detected by a rabbit anti-LC3 antibody that reacts with both LC3-I and LC3-II. The MDRV σNS protein was detected by a polyclonal antibody against the MDRV σNS protein. As shown in Fig. 2a, an obvious increase in the levels of LC3-II in MDRV-infected cells was seen and subsequent decline in the course of virus infection at 36 h post-infection (hpi) in comparison with those in mock group. An apparent increase in level of LC3-II was observed in rapamycin-treated cells and reached a maximum level of LC3-II at 36 hpi. The level of LC3-II was increased dramatically in rapamycin-treated MDRV-infected cells and reached a maximum level of LC3-II at 24 hpi, suggesting that rapamycin enhances MDRV-triggered autophagy in DF-1 cells. Chloroquine can block fusion between autophagosomes and lysosomes. As expected, the levels of LC3-II in both chloroquine-treated and chloroquine-treated MDRV-infected cells were gradually accumulated during the time course. In contrast, 3-methyladenine can inhibit the formation of autophagy vacuoles, and eventually resulted in marked reduction in the LC3-II level (Fig. 2a). The LC3-II level was rarely detectable in 3-methyladenin-treated and MDRV-infected cells, inactivated MDRV-infected cells, and mock-infected cells (Fig. 2a).

To further investigate MDRV-induced autophagy via inactivation of mTOR complex 1 (mTORC1), the levels of total mTOR and phosphorylated form of mTOR in each group were examined by Western blot assays. Results reveal that the level of p-mTOR (Ser2448) was decreased in MDRV-infected DF-1 cells as compared to mock-infected cells (Fig. 2b). Taken together, the results demonstrate that MDRV induces autophagy in DF-1 cells with the concomitant decrease in the phosphorylated form of mTOR.

**Fig. 3:**
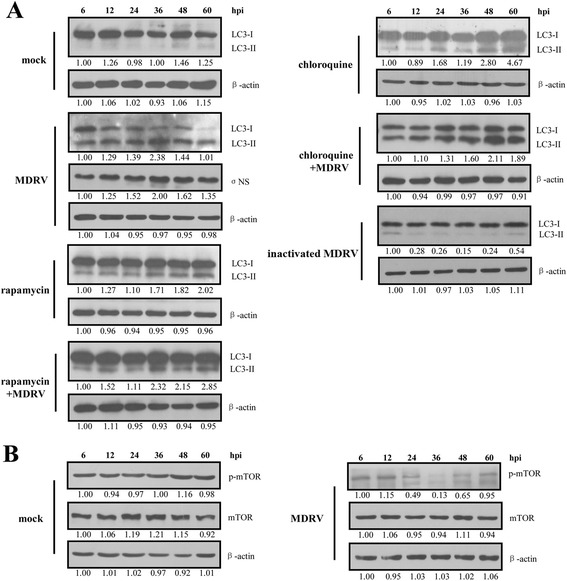
MDRV induces autophagy in MDEF cells accompanied with suppression of mTOR phosphorylation. Cells were cultured in 6-well plate and infected with the YB strain of MDRV at an MOI of 1. Samples of each group were collected at indicated time points followed by Western blot analysis of LC3 I/II (**a**), mTOR (**b**), p-mTOR (**b**), and β-actin (**a**, **b**). The activation and inactivation folds in each treatment indicated below each lane were normalized against those at 6 h. The proteins levels were normalized to those for β-actin. The levels of indicated proteins at 6 h were considered 1 fold. Similar results were obtained in three independent experiments

To further confirm MDRV-induced autophagy, the LC3-II level in MDRV-infected MDEF cells was examined. We observed that similar trend was seen in MDRV-induced autophagy as that of DF-1 cells (Fig. 3a). The results show that rapamycin and chloroquine treatments followed by MDRV infection induced a marked increase in LC3-II levels. Data presented in Fig. 3b reveal that MDRV induces autophagy in MDEF cells with the concomitant decrease in the phosphorylated form of mTOR. Collectively, MDRV is able to trigger autophagy in both DF-1 and MDEF cells via suppression of mTOR phosphorylation.

### MDRV-induced autophagy benefits virus production

**Fig. 4:**
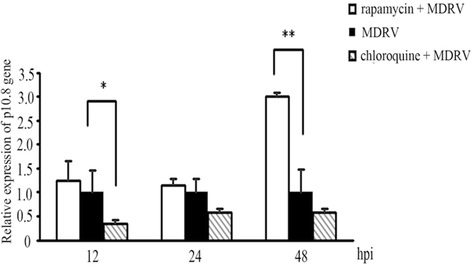
Autophagy regulators rapamycin and chloroquine affect virus yield. DF-1 cells cultured in 6-well plate were pretreated with rapamycin (100 nM) and chloroquine (50 μM) or mock-treated for 1 h, followed by MDRV infection with at an MOI of 1. Relative mRNA level of the p10.8 gene in MDRV-infected DF-1 cells was determined with real-time RT-PCR (*p < 0.05; **p < 0.01), and the mRNA levels of the p10.8 gene of MDRV was normalized relative to that of the housekeeping GAPDH gene. All the data shown represent the mean ± SD calculated from three independent experiments

**Fig. 5:**
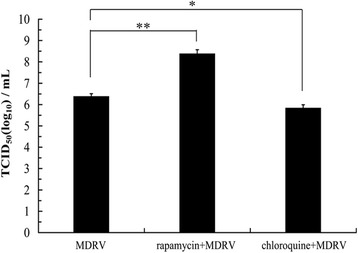
Rapamycin increases virus yield. DF-1 cells were pretreated with rapamycin (100 nM) and chloroquine (50 μM) for 1 h followed by infection with MDRV at an MOI of 1. All the data shown represent the mean ± SD calculated from three independent experiments

Several viruses have been reported to possess the ability to affect viral replication via induction of autophagy [14, 20–22]. To study whether autophagy induction during MDRV infection affects virus replication, the effects of rapamycin and chloroquine on MDRV replication were measured to study the impact of MDRV-induced autophagy on virus yield. Both rapamycin and chloroquine treatments had no effects on cell viability at the concentrations used (data not shown). As shown in Fig. 2A, DF-1 cells pretreated with rapamycin or chloroquine followed by MDRV infection exhibited increased levels of LC3-II. Data presented in Fig. 4, p10.8 transcripts were significantly increased in MDRV-infected DF-1 cells after treatment with rapamycin at 48 hpi while p10.8 transcripts in MDRV-infected DF-1 cells were reduced after treatment with chloroquine at 12 hpi. Furthermore, we collected MDRV-infected cell supernatants from rapamycin-, chloroquine- or mock-treatments to assess virus titers. Virus titers were measured with Reed-Muench method [27]. Virus titers of each treatment are shown in Fig. 5. The mTORC1 inhibitor, rapamycin, increased virus yield while chloroquine decreased virus yield. Taken together, MDRV-induced autophagy appears to benefit its own replication.

### The MDRV σNS nonstructural protein is involved in MDRV-induced autophagy

**Fig. 6:**
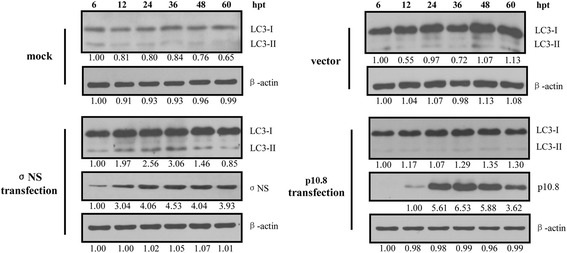
The MDRV σNS nonstructural protein is involved in MDRV-induced autophagy. DF-1 cells were cultured in 6-well plates and transfected with plasmids containing the respective p10.8 and σNS genes of MDRV. Mock-transfected and vector-transfected cells were used as controls. Samples were collected at indicated time points and analyzed with Western-blot assay using specific antibodies against LC3 I/II and β-actin. The MDRV p10.8 (*left panel*) and σNS (*right panel*) proteins were detected with rabbit polyclonal antibodies. The activation and inactivation folds in each treatment indicated below each lane were normalized against those at 6 h. The proteins levels were normalized to those for β-actin. The levels of indicated proteins at 6 h were considered 1 fold. Similar results were obtained in three independent experiments

Having demonstrated that MDRV is able to induce autophagy, we next wanted to explore which viral protein is involved in MDRV-induced autophagy. As shown in Fig. 6 (left panel), the increased levels of LC3-II was seen in the pCI-neo-flag-σNS-transfected DF-1 cells 12 h post transfection. The levels of LC3-II were not altered in the pCI-neo-flag-p10.8-transfected DF-1 cells (Fig. 6, right panel). Our results reveal that the MDRV σNS nonstructural protein plays an important role in MDRV-induced autophagy.

## Discussion

Autophagy is an important physiological process in eukaryotic cells to degrade and recycle endogenous aged bio-macromolecules, resist exogenous pathogens and maintain cellular stability. Many viruses have been reported to be able to induce autophagy [20–22, 28, 29]. The interplay between autophagy and virus is complicated and has been well studied in current molecular virology-associated research field. As mentioned above, the ARV p17 nonstructural protein induces autophagy via activation of PI3K/AKT/mTOR, PTEN, AMPK, and PKR-eIF2α signaling pathways [14]. Autophagy induced by MDRV remains largely unknown. We proposed that as in the case of a member of the family *Reoviridae*, MDRV might induce autophagy and the MDRV nonstructural proteins may play an important role in MDRV-induced autophagy. In addition, heat-inactivated MDRV could not induce autophagy in DF-1 cells, indicating that MDRV-triggered autophagy might be associated with viral replication or stimulators produced from live virus. This study explores whether the MDRV p10.8 and σNS nonstructural proteins are involved in MDRV-induced autophagy. Previous studies have shown that the MDRV p10.8 protein induces apoptosis and enters the nucleus for gene regulation [30, 31]. The ARV σNS protein possesses ssRNA-binding ability and is possibly associated with the assembly of viral mRNA, viral replication and morphogenesis [32–34]. In this work, we found that MDRV induces autophagy in MDEF and DF-1 cells via suppression of mTOR phosphorylation and the MDRV σNS nonstructural protein functions as a positive regulator of autophagy.

Several signal transduction pathways are involved in autophagy induction. The PI3K/AKT/mTOR pathway is reported to negatively regulate autophagy [13]. As mentioned above, the ARV p17 nonstructural protein induces autophagy via activation of multiple signaling pathway [14], including the PI3K/AKT/mTOR pathway. mTOR is a kind of serine/threonine kinase for regulation of cell growth, cell autophagy and cell cycle, which is divided into mTORC1 and mTORC2 [35], and mTORC1 participates in the regulation of autophagy [36]. Under normal circumstances, the phosphorylated mTOR suppresses autophagy through interaction with Atg13 and ULK1. Dephosphorylation of mTOR in turn activates autophagy process and formation of autophagosome [37]. In this study, the decreased levels of p-mTOR in MDRV-infected cells are correlated with an increase in LC3-II levels, suggesting that MDRV YB strains induces autophagy in DF-1 and MDEF cells via suppression of the mTORC1 pathway. The precise mechanisms by which the MDRV σNS nonstructural protein induces autophagy remain to be explored.

In general, LC3-II significantly increases when autophagy occurs, which is a gold standard for detection of autophagosome formation. In the present study, MDRV infected-MDEF and DF-1 cells exhibited a marked increased levels of LC3-II and gradual decrease until to 60 hpi, suggesting that MDRV YB strain induce long-term autophagy in MDEF and DF-1 cells. An apparent increase in level of LC3-II was observed in rapamycin-treated cells. Since chloroquine can block fusion between autophagosomes and lysosomes, the levels of LC3-II in both chloroquine-treated and chloroquine-treated MDRV-infected cells were gradually accumulated during the time course. Consistent with an earlier study, cells treated with chloroquine followed by ARV infection decreased virus yield [22]. In contrast to rapamycin-induced higher levels of LC3-II, which increase virus yield, chloroquine induces an increase in LC3-II levels but decreases virus yields. Since chloroquine can inhibit endosomal acidification leading to inhibition of viral infection [38–41], this may explain why chloroquine inhibits fusion between autophagosomes and lysosomes, thereby increasing LC3-II levels but decreasing virus yield. Although virus infection can trigger autophagy is well known, effects of autophagy on the mechanism of viral replication vary in different types of the viruses [20–22, 28, 29, 42, 43]. Our results of qRT-PCR and TCID_50_ assays revealed that MDRV-induced autophagy increases virus yield. This finding is similar to the case in ARV-induced autophagy benefiting virus replication [14, 22]. Like ARV, MDRV appears to evolve mechanisms that alter the physiology of host cells during infection to increase its own replication.

## Conclusions

In summary, our present results suggest that MDRV induces autophagy in MDEF and DF-1 cells benefiting MDRV replication. We report for the first time that the MDRV σNS nonstructural protein plays an important role in MDRV-induced autophagy. This study provides novel insights into the interplay of MDRV and host cells and highlights the potential role of autophagy in pathogenesis of MDRV.

## Abbreviations

AMPK: Adenosine monophosphate-activated protein kinase
ARV: Avian reovirus
DF-1: Immortalized chicken embryo fibroblast
dsRNA: Double-stranded RNA
eIF2α: Eukaryotic initiation factor 2α
HRP: Horseradish peroxidase
LC3-II: Microtubule associated protein 1 light chain 3 type II
MDEF: Primary Muscovy duck embryo fibroblasts
MDRV: Muscovy duck reovirus
MOI: Multiplicity of infection
mTOR: Mammalian target of rapamycin
mTORC1: mTOR complex 1
mTORC2: mTOR complex 2
NLS: Nuclear localization signal
ORF: Open reading frame
PI3K: Phosphoinositide 3-kinase
PKR: Protein kinase R
PTEN: Phosphatase and tensin homolog deleted on chromosome ten
TCID_50_: 50% tissue culture infective dose
TEM: Transmission electron microscopy
